# Examining electroencephalogram signatures of people with multiple sclerosis using a nonlinear dynamics approach: a systematic review and bibliographic analysis

**DOI:** 10.3389/fncom.2023.1207067

**Published:** 2023-06-29

**Authors:** Christopher Ivan Hernandez, Shaida Kargarnovin, Sara Hejazi, Waldemar Karwowski

**Affiliations:** Computational Neuroergonomics Laboratory, Department of Industrial Engineering and Management Systems, University of Central Florida, Orlando, FL, United States

**Keywords:** chaos, electroencephalogram, nonlinear dynamics, multiple sclerosis, complexity analysis

## Abstract

**Background:**

Considering that brain activity involves communication between millions of neurons in a complex network, nonlinear analysis is a viable tool for studying electroencephalography (EEG). The main objective of this review was to collate studies that utilized chaotic measures and nonlinear dynamical analysis in EEG of multiple sclerosis (MS) patients and to discuss the contributions of chaos theory techniques to understanding, diagnosing, and treating MS.

**Methods:**

Using the preferred reporting items for systematic reviews and meta-analysis (PRISMA), the databases EbscoHost, IEEE, ProQuest, PubMed, Science Direct, Web of Science, and Google Scholar were searched for publications that applied chaos theory in EEG analysis of MS patients.

**Results:**

A bibliographic analysis was performed using VOSviewer software keyword co-occurrence analysis indicated that MS was the focus of the research and that research on MS diagnosis has shifted from conventional methods, such as magnetic resonance imaging, to EEG techniques in recent years. A total of 17 studies were included in this review. Among the included articles, nine studies examined resting-state, and eight examined task-based conditions.

**Conclusion:**

Although nonlinear EEG analysis of MS is a relatively novel area of research, the findings have been demonstrated to be informative and effective. The most frequently used nonlinear dynamics analyses were fractal dimension, recurrence quantification analysis, mutual information, and coherence. Each analysis selected provided a unique assessment to fulfill the objective of this review. While considering the limitations discussed, there is a promising path forward using nonlinear analyses with MS data.

## Introduction

Multiple sclerosis (MS) is a progressive condition affecting the central nervous system (CNS). It is characterized by the formation of widespread lesions, also known as plaques, in the brain and spinal cord (Chiaravalloti and DeLuca, 2008). These plaques primarily impact the protective myelin sheath surrounding the nerve fibers, leading to a disruption in the transmission of nerve impulses (Trapp et al., 1998, 1999; Steinman, 2001). Traditionally, MS has been associated with inflammatory demyelination as the primary disease mechanism. However, recent evidence suggests that early axonal damage or loss plays a significant role, leading to permanent disability (Chelune et al., 2008). There is an ongoing debate about whether the pathology observed in the gray matter is distinct from that seen in the white matter, if it is a consequence of white matter axonal injury, or even if it shares similarities with white matter pathology (Benedict et al., 2008). Due to the widespread occurrence of plaques, MS manifests with a wide range of symptoms, including motor, cognitive, and neuropsychiatric issues (Brassington and Marsh, 1998). It is important to note that each individual with MS presents a unique combination of symptoms and disease progression.

Furthermore, cognitive impairments can manifest independently of physical disability, which makes their detection and recognition challenging (Gordon et al., 1994). This variability in symptoms and disease course complicates understanding the disease process and the development of effective treatments. While the exact cause of MS remains unknown, current research suggests that immunological, genetic, and viral factors contribute to its development (Cobble, 1992). Other symptoms of MS include weakness of the limbs or changes in sensory perception, physical disability and imbalance, visual problems, dizziness, or facial paralysis (Kesselring, 2005; Cao et al., 2015; Raeisi et al., 2020). The brain, spinal cord, and CNS are all significantly affected by MS due to its aggressive myelin damage (Dachraoui et al., 2021). Various studies have explored the brain’s nature as a nonlinear dynamical system, highlighting its intricate complexities (McKenna et al., 1994; Faure and Korn, 2001; Kotini et al., 2007). These complexities span different levels of analysis, encompassing the intricate dynamics of individual neurons to the broader macro networks within the human brain (Di Ieva et al., 2015).

A complex disease like MS has numerous pathophysiological features, necessitating the implementation of reliable tools and methods for its evaluation and assessment (Dachraoui et al., 2021). The McDonald criteria are widely used for diagnosing MS. This involves the combination of known clinical features, cerebrospinal fluid (CSF) analyses, imaging techniques such as magnetic resonance imaging (MRI), and blood tests. Since there are no distinct markers for diagnosing MS, McDonald criteria and reviewing a patient’s medical history are the best approaches for an accurate diagnosis (Ömerhoca et al., 2018). When a patient experiences a neurological deficit that lasts at least 24 h, MRI with an intravenous contrast agent is used to examine how lesions are dispersed within the CNS. The aim of this procedure is to identify the temporal and spatial distribution of lesions within the CNS (Ömerhoca et al., 2018). Research and new findings have evolved the McDonald criteria over the years, with the most recent update occurring in 2017. The criteria are applied to patients with a typical clinically isolated syndrome, similar to an MS relapse, but in a person that has not been diagnosed with MS (Thompson et al., 2018).

Among all the neuroimaging methods, MRI is currently the most widely used in MS diagnosis. Although it can be a useful technique, MRI does not correspond well with clinical manifestations of disease, and it is invasive, expensive, and time-consuming (Hossain et al., 2022). Aside from considering initial symptoms, past neurological disorders, medical conditions, etc., other methods for diagnosing MS include cerebrospinal fluid (CSF) analysis, evoked potential (EP), and blood samples analysis (Ghasemi et al., 2017). Evoked potential tests, such as those for visual, brain stem auditory, and somatosensory responses, can provide information on demyelination in the optic nerve and CNS. Additionally, diagnostic assistance may be obtained through CSF analysis for myelin basic protein, immunoglobulin-gamma (IgG) determinations, and blood sample analysis to detect vitamin deficiencies (Ghasemi et al., 2017). Physicians’ experience suggests that MRI, CSF, and EP can be subjective, invasive, and time-consuming. It is suggested that results may be subjective because routine visual inspection may yield subjective results, leading to overlooking important changes that could have been prevented (Karaca et al., 2021). Furthermore, the data are analyzed on a group basis due to the small sample size, and the grouping approach cancels out individual variations (Twose et al., 2020).

Electroencephalography (EEG) analysis could serve as the basis of a diagnostic method for monitoring organ-level changes in brain activity associated with MS; even structural changes due to MS that are not detectable by imaging techniques can be detected by EEG analysis (Carrubba et al., 2012). EEG is a non-invasive, painless, and cost-effective method for identifying brain diseases (Torabi et al., 2017). Many illnesses are associated with irregular EEG patterns. EEG signals can thereby be used to detect seizures, monitor cognitive activities, investigate the effects of sleep, and study Parkinson’s and Alzheimer’s diseases, among others (Sanei and Chambers, 2008).

In terms of signal characteristics, EEG has chaotic behavior because the amplitudes of EEG signals fluctuate in a seemingly random manner with respect to time (Rodriguez-Bermudez and Garcia-Laencina, 2015). However, in this context, “randomly” does not imply true randomness but rather refers to the evident and unpredictable nature of the amplitude changes observed in EEG signals (Natarajan et al., 2004). It is important to note that despite the appearance of randomness, EEG signals are deterministic (Pritchard and Duke, 1995). The theories of nonlinear dynamical systems and chaos theory provide frameworks to understand and describe complex behaviors exhibited by deterministic systems that may appear random (Kargarnovin et al., 2023). Therefore, it is necessary to analyze EEG signals with the help of appropriate signal-processing algorithms to uncover the hidden information within them (Beniczky and Schomer, 2020). Subsequently, researchers have recognized EEG as a signal that possesses nonlinear dynamical attributes. Moreover, EEG signal characteristics were previously captured using linear and parametric methods. Even though linear methods (e.g., fast Fourier transform, wavelet transform, and autoregressive models) have produced reasonable results, they are incapable of extracting EEG’s underlying nonlinear features and are not always accurate (Pritchard and Duke, 1995; Al-Fahoum and Al-Fraihat, 2014; Di Ieva et al., 2015; Rodriguez-Bermudez and Garcia-Laencina, 2015). According to Rodriguez-Bermudez and Garcia-Laencina (2015), nonlinear analysis has been applied to a variety of biomedical applications in recent years, such as electrocardiogram (ECG), electromyogram (EMG), electrooculogram (EOG), and magnetoencephalogram (MEG) analyses, and have been proven effective in that they are able to capture the intricate and nonlinear dynamics associated with EEG signals, quantify and characterize the nonlinear features present in nonlinear signals, differentiate between healthy and pathological EEG signals, and forecast future states of EEG signals (Rodriguez-Bermudez and Garcia-Laencina, 2015).

The purpose of this review was to collate recent studies that analyzed EEG data obtained from populations with MS using nonlinear dynamical methods and chaos theory. Accordingly, we limited the search to EEG studies that utilized chaos theory to identify indexes for brain deficits or cognitive impairments observed in people with MS. The goal was to determine if chaos theory is useful in identifying biomarkers in individuals with MS and healthy controls.

The current review is organized as follows: the “Methodology” section outlines the search strategy and inclusion and exclusion criteria used to retrieve the articles included. The “Results” section presents the literature search results, bibliometric analysis, study characteristics, and a general overview of the discussion. The “Discussion” section addresses the theoretical implications and application of nonlinear dynamics and chaos theory in the analysis of EEG in patients with MS. In the final section, “Conclusions,” we discuss future directions and developments in the application of nonlinear dynamics theories in the field of neuroscience to help patients with MS.

## Methodology

### Review standards

The systematic literature review paper followed the preferred reporting items for systematic reviews and meta-analyses (PRISMA) (Moher et al., 2009). Articles were selected based on the research questions below, and the search strategy to refine the list is identified below.

### Research questions

• RQ1: How does EEG help study MS?
• RQ2: What chaotic measures and tools have been used to analyze EEG of MS patients?
• RQ3: What are the ways in which chaos theory techniques have assisted EEG analysis in understanding and diagnosing MS?

### Search strategy

Our search was conducted in the following databases: EbscoHost, IEEE, ProQuest, PubMed, Science Direct, Web of Science, and Google Scholar. We used two major sets of keywords. We began by selecting keywords that appeared most frequently in nonlinear analysis of neurological diseases research. We used the Boolean operator “OR” between nonlinear dynamics terms to ensure all potential articles were taken into account. As a second set of keywords, we searched for “Multiple Sclerosis” to find studies using chaos theory for MS patients. The results of using a third set of keywords, such as “EEG” or “Electroencephalography,” were very limited; therefore, we decided to broaden our search and only use two major sets of terms with no restrictions regarding the publication date:

(Chaos OR Entropy OR Fractals OR “Fractal Analysis” OR “Correlation Dimension” OR “Hurst Exponent” OR “Lyapunov Exponent” OR “Phase Space” OR “Wavelet” OR “Recurrence Quantification Analysis” OR “Horizontal Visibility Graph” OR “L-Z Complexity” OR “Empirical Mode Decomposition” OR “Coherence” OR “Mutual Information” OR “Nonlinear Dynamic*” OR “Nonlinear Complex*” OR “Nonlinear Analysis” OR “Complex* Analysis” OR “Nonlinear System*”) AND “Multiple Sclerosis.”

### Criteria for inclusion and exclusion

The following inclusion criteria were applied: (a) papers written in English; (b) peer-reviewed; (c) experiments in humans; (d) studies using electroencephalograms or EEG.

**FIGURE 1:**
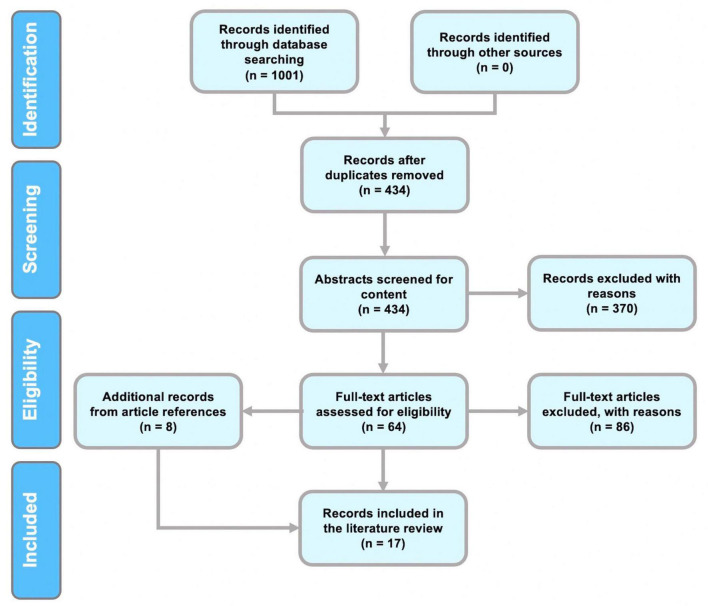
Results of our systematic literature review presented in a PRISMA flowchart.

The following papers were excluded: (a) opinions and viewpoints; (b) books and chapters (c) articles that were not related to the research questions; (c) studies that did not present original research; (d) articles that aimed to answer mental health concerns with EEG analysis; (e) articles that discussed the development of the feature extraction method; (f) studies on brain disorders and diseases such as Parkinson’s, epilepsy, seizure, schizophrenia, Alzheimer’s disease, autism, and depression; (g) studies utilizing data collection methods other than EEG. The results of the PRISMA search are shown in Figure 1.

### PRISMA chart

#### Data collection and reporting

A summary of relevant information is presented in Supplementary Table 1, which displays the study category, nonlinear dynamical analysis, number of participants, the number of EEG channels, experiment, and major findings.

## Results

### Literature search

The review was conducted in accordance with PRISMA guidelines (Moher et al., 2009). A flowchart of the procedures for identifying, screening, and selecting studies for this review is given in Figure 1. Initially, 1,001 papers were identified in the first step of identification. Removal of duplicates resulted in 434 articles. Selection of relevant scientific articles from the remaining 434 papers was performed by referring to predefined inclusion and exclusion criteria.

### Large-scale bibliometric analysis

Our bibliometric analysis was conducted with VOSviewer software (van Eck and Waltman, 2010). VOSviewer’s bibliometric network of maps can be used for visualizing and analyzing trends in publications in a particular field. Using VOSviewer, one can create a network of scientific publications, scientific journals, maps of co-authorship, countries, and keyword co-occurrences. It is possible to change the frequency of keywords and remove nonessential keywords as needed. Data mining can also be done using VOSviewer software, as well as mapping and grouping of articles from research databases (Xie et al., 2020).

**FIGURE 2:**
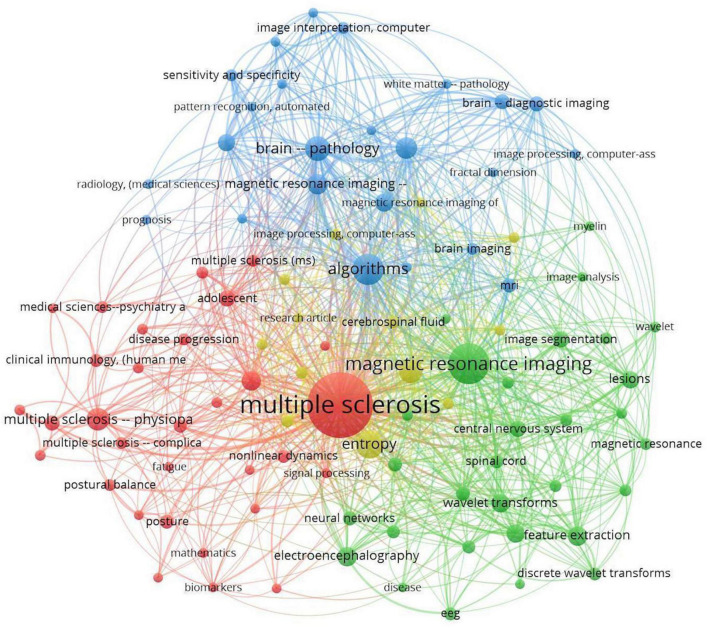
Network visualization of keyword co-occurrence.

Each database result was downloaded in.ris format and then combined into one file using Zotero’s citation management software. The.ris file exported from Zotero was used as an input file in VOSviewer after duplicates were removed and citations were refined. In the co-occurrence of keywords analysis, the minimum number of occurrences of a keyword was set to 5. Among 1,812 keywords, 108 met the threshold. In accordance with the criteria selected, VOSviewer software provided a network visualization graph of the analysis of the keywords (Figure 2). A number of keywords that were more general than technical, such as human, male, female, and adult, were excluded from the analysis.

Items are labeled and circled according to their weight. Items with higher weight have a larger label and circle. Links are co-occurrences between two keywords, and two keywords are more related if they are located close together. A numerical value represents the strength of each link in the VOSviewer network. Stronger links have a higher value. The value is calculated by counting the number of publications in which two keywords appear together.

The co-occurrence analysis graph presented in Figure 2 shows that all keywords are well related to other relevant keywords, with MS, magnetic resonance imaging, and algorithms having the highest occurrence frequency.

Table 1 shows the top 20 keywords and the number of their occurrences and total link strength.

**TABLE 1:** Top nineteen keywords with their occurrences and total link strengths.

Keyword	Weight (occurrences)	Total link strength
Multiple sclerosis	130	658
Magnetic resonance imaging	57	385
Algorithms	35	311
Brain – pathology	24	198
Entropy	30	192
Multiple sclerosis – pathology	19	177
Brain	22	152
Magnetic resonance imaging – methods	17	149
Reproducibility of results	13	119
Lesions	13	109
Feature extraction	15	108
Multiple sclerosis – diagnosis	16	108
Wavelet transforms	14	101
Machine learning	14	100
Multiple sclerosis – physiopathology	21	99
Image segmentation	11	93
Fractals	14	88
Brain – diagnostic imaging	10	84
Central nervous system	11	81
Multiple sclerosis – diagnostic imaging	10	80

As shown in Figure 2, the term “Multiple Sclerosis” has the largest circle and label and is located at the center of all keywords, indicating that MS is the primary focus of the current literature review.

**FIGURE 3:**
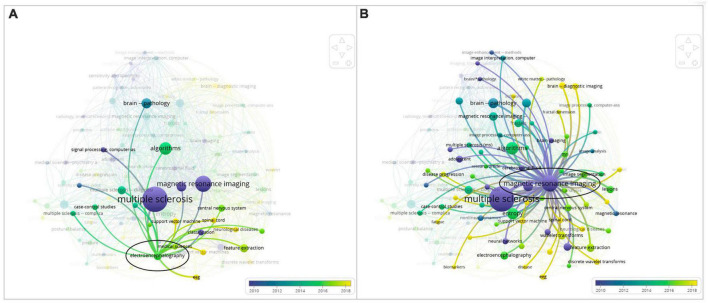
Side-by-side comparison: **(A)** overlay map of the keyword “electroencephalography” and **(B)** overlay map of the keyword “magnetic resonance imaging”.

The proximity of “Multiple Sclerosis” to “Electroencephalography” shown in Figure 3A, which is an overlay graph, indicates that “Electroencephalography” has occurred frequently with “Multiple Sclerosis” in this literature review. In general, “EEG” and “Electroencephalography” are associated with keywords such as classification, feature extraction, and machine learning.

In addition, in Figure 3, different keywords are visualized based on publication year. Each circle represents a certain period of time. In the bottom right corner of the map, a color bar indicates how scores are mapped to colors. In this instance, light green and yellow represent the most recent years, whereas dark blue and purple represent the early years of research in this field. This kind of map can be used to identify research gaps and trends easily.

When comparing the keywords “Electroencephalography” and “Magnetic Resonance Imaging,” which occur most frequently with “Multiple Sclerosis,” “Electroencephalography” has a yellow color corresponding to years 2018 and later. In Figure 3B, “Magnetic Resonance Imaging” has a purple color corresponding to years 2010 and before. Clearly, the focus of research in recent years in relation to the diagnosis of MS has shifted to electroencephalography techniques. The smaller circle and label of “Electroencephalography” support the observation that EEG is a developing research field for MS therapy.

### Study characteristics

The sample size of the studies ranged from two participants to 100 participants. The mean, mode, median, and standard deviation of the participant numbers are 33.35, 20, 29, and 23.75, respectively. The selected studies were published between 2000 and 2022. All studies included MS patients and healthy controls. Between studies, we found two distinct types of experimentation: resting-state experiments and task-based experiments. We have therefore divided the discussion into these categories. The papers that discussed details about their experiments focused on performance in attentional, visual, cognitive, or motor tasks. There were only five studies published on nonlinear analysis of EEG of MS patients before 2015, but after 2015 there was an increase in the number of publications in this field.

### Overview of the review

To answer research questions 1–3, the role of EEG in MS studies must first be understood; therefore, we describe the importance of EEG in MS research (RQ1). We then give a brief introduction to nonlinear dynamical methods utilized for EEG analysis of MS patients (RQ2). Finally, we discuss how nonlinear dynamics techniques in different experimental settings have been helpful in MS EEG studies for understanding, diagnosing, and treating MS (RQ3).

## Discussion

**FIGURE 4:**
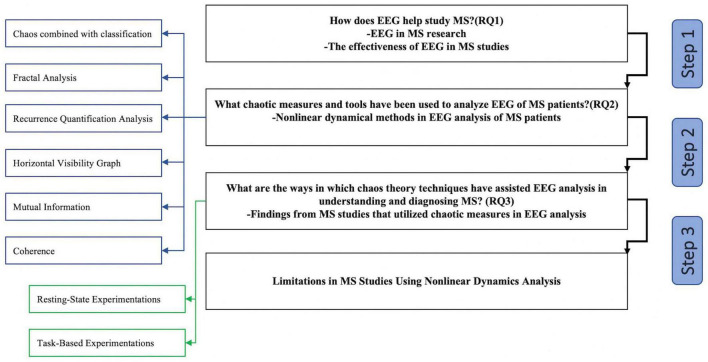
Overview of the “Discussion” section.

The “Discussion” section is divided into four major sections, as illustrated in Figure 4. Readers can see how the information in the “Discussion” section is organized by referring to the flowchart.

### EEG in MS research

As previously mentioned, cognitive deficits are some of the frequent symptoms of MS, affecting 40 to 65% of patients (Hansen et al., 2015; Korakas and Tsolaki, 2016). Information processing speed, attention, working memory, and verbal and visual memory are among the domains affected by cognitive dysfunction in MS (Prakash et al., 2008; Amato et al., 2010; Hansen et al., 2015; Korakas and Tsolaki, 2016). Cognitive impairments caused by MS are believed to be caused by white matter lesions and disruptions in fiber tracts critical for cortical connectivity (Calabrese et al., 2009; Harrison et al., 2010; Llufriu et al., 2017). Even though this is a viewpoint that is commonly accepted today, MRI results do not always coincide with disabilities shown in cognitive test results. MS cognitive dysfunction may be addressed by advanced imaging techniques, but these methods are often unavailable for regular medical evaluations. Similarly, MRI techniques such as voxel-based morphometry and diffusion tensor imaging-based fiber tracking are currently used to investigate changes in brain connectivity from anatomical aspects. These techniques are mainly applied to finding information regarding the underlying anatomical structures of large, unidirectional fibers rather than obtaining information about millisecond-level functional connectivity interactions (Cercignani et al., 2002; Audoin et al., 2007; Cader et al., 2007; Reich et al., 2007). Considering the shortcomings of neuroimaging techniques such as MRI, recent studies suggest that brain oscillatory signals observed in EEG can aid the diagnosis of cognitive deficits and facilitate the identification of pathological conditions (Hardmeier et al., 2012; Tewarie et al., 2013; Babiloni et al., 2016). For example, studies examining resting-state EEG parameters have demonstrated that an increase in slow-wave power (theta power, 4–7 Hz) and a decrease in fast-wave power (beta power, 13–30 Hz) may lead to reduced attentional control. Accordingly, an increase in the theta/beta ratio indicates a decline in attention and information-processing speed (Keune et al., 2019).

### Nonlinear dynamical methods in EEG analysis of MS

**FIGURE 5:**
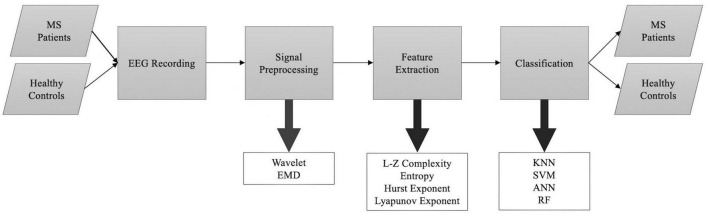
A general outline of the methodology used in studies included in this review proposing automated algorithms to separate MS patients from healthy controls.

In this section, we delve into a crucial aspect of our research by summarizing the nonlinear dynamical analysis techniques utilized in the examination of EEG signatures in individuals with MS. By exploring these techniques, we address our second research question (RQ2), which focuses on the specific chaotic measures and tools employed to analyze EEG data in MS patients. By providing this detailed overview, we aim to offer a comprehensive understanding of the methodologies employed in previous studies, which not only aids in answering our research question but also allows us to evaluate the existing body of knowledge in the field. These nonlinear analysis methods serve as valuable tools in unraveling the intricate dynamics and complexities present within EEG signals of MS patients, ultimately contributing to a more comprehensive understanding of the neurophysiological manifestations of MS. Figure 5 shows the general flowchart of the selected studies.

#### Chaos theory combined with classification approaches

A variety of machine learning models to diagnose MS have been developed using MRI data, fMRI data, EEG data, etc. Since the symptoms, severity, and progression of MS vary enormously among individuals, each patient’s prognosis and subsequent treatment decisions should be tailored to their initial conditions. A machine learning algorithm can enable the search and analysis of large datasets about potential biomarkers to help find a clinically useful course of treatment (Hossain et al., 2022).

Seven articles used feature extraction, feature selection, and feature classification in their studies (Ahmadi and Pechenizkiy, 2016; Torabi et al., 2017; Kotan et al., 2019; Raeisi et al., 2020; Karaca et al., 2021; Karacan et al., 2022; Mohseni and Moghaddasi, 2022).

To gain a comprehensive understanding of the methods employed, Table 2 provides a detailed overview of the feature extraction, selection, and classification methods each article employed, along with the nonlinear analyses used to analyze the data. We have also provided a list of definitions related to the concepts of chaos and complex systems in Table 3 as a reference for common terminology. This comprehensive summary highlights the diverse approaches adopted in the studies. The following is a description of the chaotic measures used in studies combining nonlinear dynamical methods with classification:

**TABLE 2:** Feature extraction, selection, and classification methods used.

References	Feature extraction and selection	Feature classification	Chaotic metric employed
Torabi et al., 2017	*T*-test and Bhattacharyya	K-nearest neighbors (kNN) and support vector machine (SVM)	Lyapunov exponents, approximate entropy. Sample entropy, Hurst exponent, fractal dimensions, and L-Z complexity
Ahmadi and Pechenizkiy, 2016	N/A	N/A	Horizontal visibility graph
Karacan et al., 2022	Python MNE library (peak to peak, mean, standard deviation, skewness, kurtosis, root mean square, sample entropy, approximate entropy, Higuchi, and Katz fractal dimensions) and recursive feature elimination (RFE)	Multilayer perceptron (MLP), k-nearest neighbor (kNN), and support vector machine (SVM)	Wavelet transform
Mohseni and Moghaddasi, 2022	Sample entropy (SE), Katz fractal dimension, Higuchi fractal dimension, power spectral density (PSD), Welch’s method, and fast Fourier transform (FFT) Ant colony optimization method (m-ACO)	Support vector machine (SVM)	Sample entropy, Higuchi fractal, dimension, and Katz fractal dimension
Kotan et al., 2019	Hurst exponent, Higuchi fractal dimensions, and kurtosis parameters	k-nearest neighbor (kNN), multilayer perceptron (MLP), and random forest	Empirical mode decomposition (EMD), Hurst exponent, and Higuchi fractal dimensions
Karaca et al., 2021	Wavelet coefficients from continuous wavelet transform (CWT)	Support vector machine (SVM), ensemble bagged tree, K-fold cross-validation algorithm, and k-nearest neighbor (kNN)	Wavelet transform
Raeisi et al., 2020	ReliefF algorithm	k-nearest neighbor (kNN)	Bivariate empirical mode decompositions (BEMD) and phase coherence

**TABLE 3:** Terms associated with chaos theory.

Bifurcation	A phenomenon whereby the topological characteristics of a dynamical system’s solutions change due to a minor alteration of a system’s parameter (i.e., an oscillation state transitions to a chaotic state) (Tsumoto et al., 2012).
Phase space	A group of potential system states. Phase space is essential for the analysis of dynamical systems (Pham, 2020).
Attractor	A region in the d-dimension phase space. This region consists of points with characteristics that are used to measure a dynamical system and identify a specific region in phase space. As a system commences with unique characteristics, a point begins at a distance. Over time, it approaches the attractor, hence why these regions of space are termed “attractors” (Pham, 2020).
Fractals	Complicated sets with a non-integer dimensions that exhibit statistical self-similarity across various scales (Aguirre et al., 2009).
Correlation dimension	Represented as CD or D2, the correlation dimension is the fractional dimensionality of a fundamental process concerning the geometric reconstruction in embedded phase space. The values of the correlation dimension range between zero and the embedded dimension. A value greater than one typically represents a chaotic system (Ma et al., 2018).
Fractal Dimension	The quantitative measurement of the irregularity of an object. It shows the capability of the set to fill the Euclidean space in which it exists, and it quantitatively describes fractal properties (Xu et al., 1993).

#### Wavelets

Wavelets were developed in the 1980s as a substitute for Fourier transform. Currently, wavelets are used for analyzing signals, image processing and recognition, turbulence, etc., in a variety of different fields such as physics, fluid dynamics, and others (Pavlov et al., 2012). Continuous wavelets are useful in explaining nonstationary signals where their spectral composition and statistical characteristics fluctuate over time. According to Pavlov et al. (2012), wavelets:

• show the time-frequency structure of signals, which permits localization of certain features in time and frequency domains;
• allow efficient analysis of short-term time series that have a small population of characteristic oscillation periods;
• allow for the selection of the basic functions into which the signal is expanded, allowing for the consideration of data-specific characteristics; and
• ensure efficient analysis of noisy data.

There is a smoothing effect in MS data processing that poses a challenge. Large signals can exhibit these localized features as peaks or noise. To separate the true features from noise without bias, a few studies (Karaca et al., 2021; Karacan et al., 2022) have used wavelet transforms for MS EEG to capture these localized features in different resolutions. The continuous wavelet transform (CWT) is a mathematical operation that involves multiplying the original signal by a wavelet function ψ that is scaled and shifted across all time. The CWT coefficients are obtained using Equation 1 where *f* (*t*) represents the original signal, *a* is the scaling coefficient, and *b* is the shift coefficient (Karaca et al., 2021).


(1) $${\text{W}}_{\text{f}}\,\left(a,b\right)=\int_{-\infty }^{+\infty }\text{f}\,\left(\text{t}\right)\,\bar{\psi_{a,b}}\,\left(t\right)\,\text{dt}$$

The wavelet function, ψ_*a*,*b*_ (*t*), is the complex conjugate of the wavelet function and is defined as follows:


(2) $$\psi \,a,b\,\left(t\right)=\frac{1}{\sqrt{\left|a\right|}}\,\psi \,\left(\frac{t-b}{a}\right)$$

#### Empirical mode decomposition (EMD)

Nonlinear and nonstationary signals can be analyzed using the empirical mode decomposition (EMD) method. EMD decomposes nonlinear signals into intrinsic mode functions (IMFs). IMFs refer to output signals whose amplitudes and frequencies are slowly shifting. The components of the main signal can be reflected in IMFs. It is important to note that some of these components are of limited use, while others reflect the characteristics of the original signal. Thus, selecting the appropriate IMFs is crucial (Kotan et al., 2019).

Empirical mode decomposition (EMD) breaks down the non-periodic and non-stationary signal, *X*_*DFT*_ (*t*), into a limited set of IMFs and a residue, *r*_*N*_ (*t*), as shown in Equation 3.


(3) $$X_{D\,F\,T}\,\left(t\right)=\sum_{j=1}^{N}I\,M\,F_{j}\,\left(t\right)+r_{N}\,\left(t\right)$$

In Equation 3, N represents the total number of IMFs [*IMF*_*j*_ (*t*) denotes the *j^th^* IMF], and *r*_*N*_ (*t*) corresponds to the residue obtained by selecting N IMFs (de Santiago et al., 2018).

The IMFs must satisfy two key conditions: firstly, the number of extremes (maxima and minima) and the number of zero crossings should either be equal or differ by no more than one throughout the dataset. Secondly, the mean value of the envelope formed by the local maxima and the envelope formed by the local minima should be zero at each point. In essence, the IMFs exhibit nearly periodic behavior with a mean of zero (de Santiago et al., 2018).

To decompose the signal, *X* (*t*) = *X*_*DFT*_ (*t*), into its constituent IMFs, the following four-step method is employed:

1) Identify all extreme points (maxima and minima) of the signal, *X* (*t*).
2) Create the upper and lower envelopes (*UE and LE*) by interpolating the maxima and minima using a cubic spline.
3) Calculate the mean value, $\left(t\right)=\frac{U\,E\,\left(t\right)+L\,E\,\left(t\right)}{2}$.
4) Obtain the signal, c(t), by subtracting the mean from the original signal: *c* (*t*) = *X* (*t*) − *M* (*t*).

This process is repeated iteratively until the resulting signal, c(t), satisfies the criteria of an IMF. At this stage, *c* (*t*) becomes *IMF*_1_, and the residue, *r* (*t*) = *X* (*t*) − *c* (*t*), replaces the original input signal for the subsequent step (1). Therefore, *X* (*t*) = *r* (*t*) (de Santiago et al., 2018).

In one of the selected articles, Kotan et al. (2019) used the EMD approach to distinguish between MS and healthy controls. Considering that one of the major causes of disability in MS patients is visual deficits, Raeisi et al. (2020) proposed a method based on visual stimulation and EMD for classification.

Torabi et al. (2007) employed the following feature extraction methods to find the most appropriate combination of nonlinear dynamical methods and classifier methods to differentiate MS patients from healthy subjects (Torabi et al., 2007).

#### Lempel-Ziv complexity

Lempel-Ziv (LZ) complexity is a measure used to analyze the complexity of discrete-time physiologic signals, such as frequency, number of harmonics, frequency variability of signal harmonics, and signal bandwidth (Aboy et al., 2006). The calculation of the LZ complexity steps involves graining the time series *X* (*x*_1_, *x*_2_, …, *x*_*n*_) into subsequences using a threshold value, which is always the average quantity. The signal is divided into two parts based on the threshold, assigning 1 to data larger than the threshold and 0 to data smaller than the threshold (Zhao et al., 2023).


$$T=\sum_{i=1}^{n}x_{i}\,1\leq i\leq n$$

(4) $$s_{i}=\left\{ \begin{array}{c} 0,x_{i}<T\\ 1,x_{i}\geq T \end{array}\right.$$

This graining process creates a sequence *P* ={*s*_1_, *s*_2_, …, *s*_*n*_}. Subsequences *S* and *Q* are then connected to form *SQ*. For example, if *S* = {*s*_1_, *s*_2_, …, *s*_*r*_} and *Q* = {*s*_*j*_, …, *s*_*j* + *m*_}, then *SQ* is *SQ* = {*s*_1_, *s*_2_, …*s*_*r*_, *s*_*j*_, …, *s*_*j* + *m*−1_}. If *Q* is not a subset of *SQ*, it becomes a new subsequence, and if *Q* is a subset of *SQ*, it is reconstructed as *Q* = {*s*_*j*_, …, *s*_*j* + *m* + 1_}. The number of times *Q* becomes a new subsequence is indicated as *c* (*n*). Subsequently, the complexity is normalized by the following equation:


(5) $$C=\frac{c\,\left(n\right)\,\log_{2}n}{n}$$

However, since coarse graining ignores details of the original time series, a refined graining method is developed using median and quartiles as boundary points to divide the signal into four sections:


(6) $$s_{i}=\left\{ \begin{array}{c} 0,x_{i}<l\,o\,w\,e\,r\,q\,u\,a\,r\,t\,i\,l\,e\\ 1,l\,o\,w\,e\,r\,q\,u\,a\,r\,t\,i\,l\,e\leq x_{i}<M\\ 2,M\leq x_{i}<u\,p\,p\,e\,r\,q\,u\,a\,r\,t\,i\,l\,e\\ 3,x_{i}\geq u\,p\,p\,e\,r\,q\,u\,a\,r\,t\,i\,l\,e \end{array}\right.\,1\leq i\leq n$$

*M* denotes the median. Lastly, the refined Lempel-Ziv complexity (Equation 7) can be defined as:


(7) $$C_{r}=\frac{c\,\left(n\right)\,\log_{4}n}{n}$$

Lempel-Ziv (LZ) has been applied to analyze the reactions of neurons in the primary visual cortex when exposed to various stimuli (Szczepaski et al., 2003). Also, it has been employed to evaluate the entropy of neural discharges, commonly referred to as spike trains (Amigó et al., 2004). LZ complexity has several advantages, and they are as follows:

• it can be applied to any time series;
• it can be used with nonstationary signals;
• it is a nonlinear measure that is non-parametric; and
• it is not difficult to compute.

In EEG signals, the reliance between amplitude and frequency plays an important role in the calculation since the binarization is led by slow rhythms. Thus, slow rhythms, or high amplitudes, impact the mean or median more than faster rhythms or low amplitudes (Ibáñez-Molina et al., 2015). LZ complexity is a suitable measure to aid in further understanding the complex and random nature of the EEG signals obtained from subjects with MS (Torabi et al., 2017).

#### Entropy

Entropy is a measure used to examine the ambiguity of an information source and the probability distribution of the source’s samples. Entropy can be an indicator of the complexity of a system. There are several types of entropy analyses, such as approximate entropy (ApEn) and sample entropy (SampEn) (Ma et al., 2018). Quantifying irregularity in time series is done using ApEn (Pincus, 1991). However, ApEn requires a large dataset, and if the data length is short, the method is not robust enough (Richman and Moorman, 2000). SampEn is defined as the negative logarithm of the conditional probability that two sequences of m points remain similar when m+1 is reached, with each vector being counted over all others except itself. Thus, unlike ApEn, SampEn is relatively consistent irrespective of dataset size (Richman and Moorman, 2000). Mohseni and Moghaddasi (2022) used sample entropy in the feature-extraction step of their proposed method to create an MS diagnostic tool (Mohseni and Moghaddasi, 2022). See reference Li et al. (2018) for more information on entropy, its measures, and variants.

#### Hurst exponent

This measure appraises long-term memory processes in a time series. To analyze a time sequence *X* (*x*_1_, *x*_2_, …, *x*_*n*_) with continuous values, the first step is to take the logarithm of the sequence and then perform a single differentiation of *M_i_*.


(8) $$M_{i}=\log\left(\frac{x_{i}+1}{x_{i}}\right),i=1,2,\ldots ,n-1$$

This results in a logarithm sequence. The logarithm sequence is then divided into “A” adjacent subsets, with each subset having a length of $h=\frac{\left(n-1\right)}{A}$. Within each subset, the mean value is denoted as *e_a_* and the standard deviation as *S_a_*, where *a* = 1, 2,…, A. Within each subset, the accumulated intercept of the mean for each previous k point is calculated as follows:


(9) $$X_{k,a}=\sum_{i=1}^{k}\left(M_{i,a}-e_{a}\right),k=1,2,\ldots ,h$$

The fluctuating range of each subset can be obtained by *R_a_*:


(10) $$R_{a}=\max\left(X_{k,a}\right)-\min\left(X_{k,a}\right)$$

Next is rescaling the range:


(11) $${\left(\frac{R}{S}\right)}_{h}=\left(\frac{1}{A}\right)\times \left(\frac{R_{a}}{S_{a}}\right)$$

By increasing the value of *h*, we can obtain the rescaled range of subsets with varying lengths. The Hurst exponent describes the proportional relationship between (*R*/*S*)_*h*_ and *h* as (*R*/*S*)_*h*_ = *c* × *h^HE^*, where *c* is a constant and *HE* is the Hurst exponent. Thus, the Hurst exponent can be estimated by plotting the logarithm of (*R*/*S*)_*h*_ against the logarithm of *h*. The slope of the fitted line in the plot corresponds to the Hurst exponent (Zhao et al., 2023).

The value of the exponent ranges between 0 and 1. There are three classification categories:

• H = 0.5 represents randomness with a lack of correlation;
• 0 < H < 0.5 represents an inversely related process; and
• 0.5 < H < 1 represents a correlated process with chaotic characteristics (characteristics fall on a 1/f power spectrum) (Ma et al., 2018).

Kotan et al. (2019) extracted the Hurst exponent from the collected EEG signals in MS patients to examine the self-similarity of the data.

#### Lyapunov exponent

The Lyapunov exponent is a measure used to evaluate the average exponential divergence or convergence of nearby trajectories in phase space. A positive Lyapunov exponent indicates chaos in the system (Yakovleva et al., 2020).


(12) $$k\,\left(t\right)=K\,e^{\lambda_{1}\,t}$$

In formula 12, the equation k(t) represents the measure of divergent distance, where K represents the initial distance and λ_1_ denotes the largest Lyapunov exponent (Zhao et al., 2023).

In this approach, a proper feature selection is necessary for improving modeling efficiency and laying the groundwork for subsequent steps (Wang et al., 2013). As a final step, different classification methods will be used to identify MS patients from controls, including k-nearest neighbors (KNNs), support vector machines (SVMs), artificial neural networks (ANNs), and random forests (RFs), among others (Ahmadi and Pechenizkiy, 2016; Torabi et al., 2017; Raeisi et al., 2020).

#### Fractal analysis

Fractal analysis is a measure of the complexity and self-similarity of a signal that is investigated in the phase space via the attractor dimension or other correlated parameters, and it is able to analyze information at shorter intervals when compared to other linear and nonlinear analyses (Accardo et al., 1997). More specifically, fractal analysis quantitatively assesses the roughness of neural structures, approximation of time series, and representation of patterns. It is able to differentiate different brain states in the physiopathological spectrum (Di Ieva et al., 2015). According to Accardo et al. (1997), there are several ways to calculate the fractal dimension (i.e., box counting, Walker’s Ruler, and a modified correlation dimension D2 analysis) (Mandelbrot and Mandelbrot, 1982; Pickover and Khorasani, 1986; Tirsch et al., 1991; Gonzato et al., 1998). Higuchi’s is the most widely used method for determining fractal dimension.

The calculation of Higuchi’s fractal dimension involves analyzing a time series data sequence, denoted as *X*(1), *X*(2), …, *X* (*N*), where *N* represents the total number of samples. The process begins by selecting a scale factor, *m*, which determines the length of the subseries for analysis and *k* represents the index of the subseries being analyzed. For each subseries, the cumulative length, L(m, k), is computed using a specific formula that compares the absolute differences between adjacent data points within the subseries (Porcaro et al., 2020):


(13) $$L_{m}\left(k\right)=\frac{1}{k}\left[\sum_{i=1,i\,n\,t\,\left(\frac{N-m}{k}\right)}|X\left(m+ik\right)-X\left(m_{\left(i-1\right)}k\right)|.\frac{N-1}{i\,n\,t\,\left(\frac{N-m}{k}\right)}\right]$$

*N* represents the original time series X’s length, and $\frac{N-1}{i\,n\,t\,\left(\frac{N-m}{k}\right)}$ is a factor used to normalize the function. The cumulative lengths are then averaged across all subseries to obtain *L* (*k*), the average length for the given scale factor:


(14) $$L\,\left(k\right)=\frac{\sum_{m=1}^{k}L_{m}\,\left(k\right)}{k}$$

Finally, the Higuchi fractal dimension is calculated by taking the logarithm of *L* (*k*):


(15) $$F\,D=\frac{\ln\left(L\,\left(k\right)\right)}{\ln\left(1/k\right)}\,f\,o\,r\,k=1,2,\ldots ,k_{m\,a\,x}$$

The resulting fractal dimension value represents the fractal dimension of the time series, providing insight into its complexity.

The fractal dimension is always expected to be between 1 and 2 since the dimension of a plane is 2, and the dimension of a line is 1. The fractal dimension increases when an EEG line fluctuates as more of the plane is covered (Accardo et al., 1997). A fractal dimension analysis of an MRI scan clearly shows the changes in white matter and gray matter in the early stages of MS. Clinical decision-making could be supported by the use of this approach as an early diagnostic biomarker (Di Ieva et al., 2015). Torabi et al. (2017), Kotan et al. (2019), and Mohseni and Moghaddasi (2022) extracted nonlinear features such as fractal dimensions to explore the nonlinear nature of EEG signals and sub-bands in MS patients (Torabi et al., 2017; Kotan et al., 2019; Mohseni and Moghaddasi, 2022). Porcaro et al. (2019) used fractal dimensions to analyze task-based EEG to develop a better treatment method (Porcaro et al., 2019).

#### Recurrence quantification analysis (RQA)

Recurrence is defined as stretches of long or short repeated patterns that exist in living and non-living systems. As signals become more complex, recurrence becomes more uncommon (Webber and Zbilut, 2005). Analyzing recurrence patterns requires a mathematical or quantification analysis, which paves the way for using RQA (Webber and Marwan, 2015). Five variables are considered in RQA: percent recurrence (REC) or recurrence rate (RR), percent determinism (DET), maximal length in the diagonal direction (Dmax), Shannon entropy of the frequency distribution of the diagonal line lengths (ENT), and trend (TND) (Webber and Marwan, 2015). These measures are mainly concerned with the lengths, numbers, and distributions of the diagonal lines in recurrence plots. Three additional variables were added to examine intermittency and chaos-order transitions: laminarity (LAM), the average length of vertical structures (trapping time or TT), and the maximal length of the vertical structures (Vmax). These RQA parameters can be obtained from the recurrence plots (RP) of the EEG signals using the following formulas (Khodabakhshi and Saba, 2020):


(16) $$R\,R=\frac{1}{N^{2}}\,\sum_{i,j=0}^{N}R_{i,j}$$

Where N is the total count of states and *x_i_* is the considered state.


(17) $$D\,E\,T=\frac{\sum_{l=l_{m\,i\,n}}^{N}l\,P\,\left(l\right)}{\sum_{i,j}^{N}R_{i}}$$

Here, *l*_*min*_, refers to the length of the smallest diagonal line, while *P* (*l*) represents the distribution of frequencies for different lengths (*l*) of diagonal lines. The *D*_*max*_, *ENT*, and *LAM*, respectively, are given by:


(18) $$D_{m\,a\,x}=\max\left(\left\{l_{i};i=1,\ldots ,N_{l}\right\}\right)$$

(19) $$E\,N\,T=-\sum_{l=l_{m\,i\,n}}^{N}P\,\left(l\right)\,l\,n\,P\,\left(l\right)$$

(20) $$L\,A\,M=\frac{\sum_{v=v_{m\,i\,n}}^{N}v\,P\,\left(v\right)}{\sum_{v=1}^{N}P\,\left(v\right)}$$

In this context, *P* (*v*) refers to the histogram that captures the distribution of lengths, (*v*), of vertical lines. In addition, *TT* and *V*_*max*_ are calculated by:


(21) $$T\,T=\frac{\sum_{v=v_{m\,i\,n}}^{N}v\,P\,\left(v\right)}{\sum_{v=v_{m\,i\,n}}^{N}P\,\left(v\right)}$$

(22) $$V_{m\,a\,x}=\max\left(\left\{v_{i};i=1,\ldots ,N_{v}\right\}\right)$$

Several studies have compared EEGs from patients with those from healthy controls using RQA identifiers, seeking to identify a relationship between baseline EEG and MS status (Carrubba et al., 2012, 2019). RQA is useful in various nonlinear datasets and can also be coupled with principle component analysis (PCA) (Webber and Marwan, 2015).

#### Horizontal visibility graph (HVG)

Systems in real life that have many components interacting nonlinearly usually require correlation analysis. Similarly, correlation analysis has evolved into complex network analysis (Xie et al., 2019). Finding synchronization between the signals of a neural system, such as the brain, has a critical role in characterizing the system activities and the integration of information within and across a disorder. The visibility graphs (VGs) algorithm enables the mapping of time series to complex networks. In the case of an EEG signal ${\left\{x\,\left(t\right)\right\}}_{t=1}^{N}$ with N data samples, each sample is represented as a node in a histogram-based graph. The height of each histogram bar corresponds to the value of the respective data node. Connections between nodes exist if the tops of the bars are visible. For two nodes (*t*_*i*_, *x*_*i*_) and (*t*_*j*_, *x*_*j*_), an edge is formed between them if there exists any data node (*t*_*k*_, *x*_*k*_) between (*t*_*i*_, *x*_*i*_) and (*t*_*j*_, *x*_*j*_) that satisfies the convexity criterion (Lacasa et al., 2008):


(23) $$\frac{{\text{x}}_{\text{i}}-{\text{x}}_{\text{k}}}{t_{k}-t\,i}>\frac{{\text{x}}_{\text{i}}-{\text{x}}_{\text{j}}}{t_{j}-t_{i}},t_{i}<t_{k}<t_{j}$$

The horizontal visibility graphs (HVGs) algorithm is a modified version of the VG algorithm. In HVG, two data nodes (*t*_*i*_, *x*_*i*_) and (*t*_*j*_, *x*_*j*_) are considered to have horizontal visibility if they meet the condition stated in Equation 24 (below):


(24) $$x_{i},x_{j}>x_{k},t_{i}<t_{k}<t_{j}$$

Therefore, (*t*_*k*_, *x*_*k*_)represents a data node located between (*t*_*i*_, *x*_*i*_) and (*t*_*j*_, *x*_*j*_). The complex network is represented by an adjacency matrix *A* = (*a*_*ij*_)_*N* × *N*_, where *a*_*ij*_ = 1 if nodes *t_i_* and *t_j_* are connected, and *a*_*ij*_ = 0 if they are not (Ahmadi and Pechenizkiy, 2016).

The application of synchronization measures, such as VGs and HVGs, to capture any intrinsic interactions between two-time series that are not stationary can thus prove beneficial (Dong et al., 2019). Thus, synchronization measures that capture the key features of the system can significantly contribute to our understanding of it. Based on EEG signals from healthy controls and MS patients, the authors suggest the successful application of the HVG method to construct a synchronization matrix of the brain network (Ahmadi and Pechenizkiy, 2016).

#### Mutual information

Mutual information is a measure of nonlinear dependence between two random variables. It is reliant on the random variable that diverges from random chance. If both random variables are independent, the joint entropy of their variables equals the sum of the marginal entropies. Dependence is observed when the joint entropy is less than the sum of the marginal entropies. If the joint distribution of two random variables is given by *p* (*X*, *Y*) and their factored marginal distributions are *p* (*X*) and *p* (*Y*), the formula for mutual information, *I* (*X*; *Y*) is as follows (Moermans et al., 2018):


(25) $$I\,\left(X;Y\right)=\sum_{y\in Y}\sum_{x\in X}p\,\left(X,Y\right)\,\log\left(\frac{p\,\left(X,Y\right)}{p\,\left(X\right)\,p\,\left(Y\right)}\right)$$

The values for mutual information can range from 0 to infinity, where 0 represents total independence between the random variables, and infinity represents two correlated and continuous random variables (Smith, 2015). The effect of treatment on fatigue in MS was evaluated by calculating mutual information between the somatosensory and motor cortex in each hemisphere (Porcaro et al., 2019). Other studies have also used mutual information to evaluate and compare two EEG signals (Lenne et al., 2013; Tramonti et al., 2018).

#### Coherence

Coherence has been extensively employed across a range of research disciplines, including but not limited to time-series-based studies, physics, and image processing, for the purpose of measuring the linear synchronization between two-time series. In simple terms, coherence denotes the interrelationship of two-time series at a specific frequency. One important application of coherence is in linear filtering, particularly in the context of EEG analysis that involves the presence of noise (Lenne et al., 2013).

In this context, for EEG data, coherence calculations can determine neural population synchronization levels among different brain regions (Nunez et al., 1999). The classic expression for coherence is:


(26) $$r_{x\,y\,\omega }=\frac{s_{x\,y\,\omega }}{\sqrt{s_{x\,x\,\omega }\,s_{y\,y\,\omega }}}$$

In this equation, *s*_*xyω*_ indicates the cross-spectrum between *x* and *y* signals in the ω frequency (Jouzizadeh et al., 2021).

Coherence seeks to understand the information transfer between a known variable and an unknown variable. The measure increases with dependency between the two variables (Lenne et al., 2013). In progressive MS patients, EEG coherence shows a significant decrease between the anteroposterior and interhemispheric areas in alpha and theta bands, which correlates with cognitive dysfunction and subcortical lesion severity found on MRI results (Leocani et al., 2000; Lenne et al., 2013).

**FIGURE 6:**
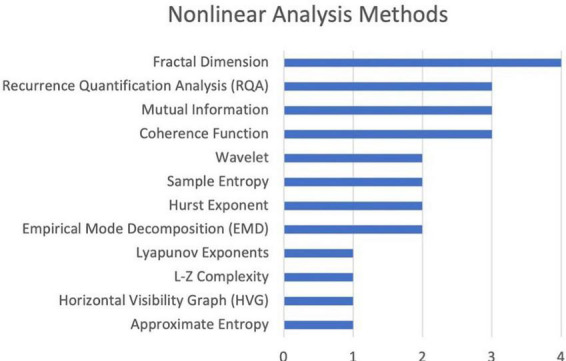
Frequency of different nonlinear analysis methods.

Figure 6 lists the name and frequency of each method used in the studies included in this review. The most common method of chaos quantification was fractal dimension analysis.

### MS studies utilizing chaotic measures: findings

As part of the first studies on MS, tasks were used to identify abnormal activity in associated brain regions or networks involved in the task. Over time, studies of resting-state functional connectivity became more prevalent (Rocca et al., 2022). In these studies, large-scale functional networks of the brain were mapped, and it was established that MS pathology impairs functional connectivity, resulting in a degradation of the anticipated network following the course of the disease. There is evidence that reduced attentional capacity may be associated with an increase in theta activity in resting-state EEG and a decrease in beta-wave activity (Keune et al., 2017). Thus, this section provides a summary of the main findings from the studies included in this literature review. These studies either used resting-state experiments or task-driven tests for visual, attentional, or cognitive impairments identified in signature EEGs of patients with MS.

#### Resting-state experiments

Kotan et al. (2019) compared three methods for selecting IMFs for EEG signals; power-based, correlation-based, and power-spectral density-based combined with Hurst exponent and Higuchi fractal dimensions. Then, three classifiers: KNN, multilayer perceptron neural networks, and RF, were employed to detect cognitively impaired and cognitively intact MS patients using the nonlinear features of Hurst exponent and fractal dimension. The authors concluded that IMF selection methods have a variable effect on accuracy based on classifier selection. The findings of their study indicate that different methods for selecting IMFs have varying effects on the classification accuracy of EEG signals collected from multiple sclerosis (MS) patients. Therefore, it is recommended to employ various IMF selection methods along with different classifiers and different types of signals to better understand their effects on classification accuracy.

Using RQA, Carrubba et al. (2019) developed a method for detecting MS from EEG signals. They assessed RQA application by statistically comparing the values of its quantifiers of EEGs from patients having MS with values from the EEGs of healthy subjects. They found that optimal embedding dimension and a time delay of 5 points maximize RQA’s ability to detect deterministic activity in EEG time series (Carrubba et al., 2019). In the MS patients, the RQA quantifier values were significantly higher than those in the healthy controls, indicating that the disease is associated with detectable changes in the EEG. Accordingly, a decrease in complexity associated with MS was observed in EEG recurrence plots (Carrubba et al., 2010). In another study by Carrubba et al. (2012), the same RQA method was employed. They proposed that EEG signals represent an instantaneous sum of contributions from several neural networks that elicit intra- and internetwork interactions. Therefore, the authors used RQA quantifiers to detect any changes from the “unknown, but certain laws” that brains without MS usually follow. The authors examined the RQA quantifiers, percent recurrence (%R), and percent determinism (%D). They observed an increase in %R in MS patients and no difference in %D in MS patients compared with healthy subjects, suggesting that %R is an indicator of nonlinearity (Carrubba et al., 2012). Therefore, due to their ability to reveal evoked potentials, %R and %D are possible indicators for MS diagnosis.

Lenne et al. (2013) also analyzed functional information about interhemispheric and intra-hemispheric cortical communication. As part of their methodology, they compared the coherence between different EEG bands and the mutual information between bipolar EEG signals between patients and healthy controls. The main outcome of cortical communication impairment in MS was a significant decrease in mutual information between brain areas. This study indicated that averaged interhemispheric mutual information in the resting state may indicate neurological dysfunction in MS patients. In addition, these findings indicate that this nonlinear measure may serve as an indicator of MS patients’ information processing deficits. Using mutual information, one can thus characterize the connectivity of brain information transmission (Lenne et al., 2013).

The results reported by Leocani et al. (2000) align closely with those reported by Lenne et al. (2013). The authors observed a reduction in coherence in EEG activity between the anteroposterior and interhemispheric areas in the cognitively impaired MS patients. Some findings revealed that there was unusual synchronization and hyperconnectivity in certain frequency bands, which could be due to the compensatory mechanisms or pathological abnormalities associated with MS. These findings emphasized that coherence analysis could be used as an indicator for cognitive dysfunction connected to multiple sclerosis (MS) while understanding changes that take place in the brain. Thus, cognitive impairment in MS is contingent on the corticocortical connection tied to the demyelination and/or the axonal loss that lies beneath the cortex in the white matter (Leocani et al., 2000).

Tramonti et al. (2018) used weighted symbolic mutual information (wSMI) to evaluate non-random joint fluctuations between two EEG signals after participants underwent gait rehabilitation. Their analysis provided strong results for understanding behavioral changes and showed that phase synchronization is correlated with functional recovery (Tramonti et al., 2018).

Carrubba et al. (2010) successfully applied chaotic measures for the diagnosis of MS. By introducing a subliminal stimulus in patients with MS and those without and through RQA quantifiers, they tested whether cognitive processing was altered in patients with MS, finding that MS patients had 27% onset responses in EEG signals and subjects without MS had 85% onset responses. Using this study’s methodology, one can assess the degree of synchronization between brain networks, which is exactly the high-level brain function they believed to be impaired (Carrubba et al., 2010).

Mohseni and Moghaddasi (2022) also employed nonlinear feature extraction and classification. Through segmenting random signals in EEG signals, they proposed a model for diagnosing MS. While the alpha, beta, and gamma sub-bands have an impact on signal analysis, the robustness of the technique with random selections of 15-to-30-second segments of the EEG signal and the detecting power of the algorithm makes it appropriate for generalization (Mohseni and Moghaddasi, 2022).

Jouzizadeh et al. (2021) applied coherence analysis to understand the functional connectivity of MS. They discovered differences in the functional connectivity between patients with MS and healthy subjects. The information transfer and increase in communication distances were observed due to the deficit in global efficiency and the increase in path size. To add, it was determined that MS has a large impact on the brain since the connectivity patterns in the frontal, parietal, and occipital regions were all affected, which indicates there would be issues with the cognitive, sensory, and visual processing areas. The authors noted that the results produced similar results to those generated by fMRI, and it was also determined that betweenness centrality and small-world propensity are effective indicators in differentiating an individual with MS from an individual without MS (Jouzizadeh et al., 2021).

Buyukturkoglu et al. (2017) aimed to investigate the functional connectivity patterns in the brain related to fatigue in multiple sclerosis (MS) patients. They focused on the concept of functional connectivity, which refers to the degree of coherence or synchronization between different brain regions. The main finding of the study was that a potential EEG-Neurofeedback system for MS fatigue should train patients to voluntarily decrease coherence in the beta frequency band between homologous temporoparietal cortices. By targeting and modulating this specific brain organization feature, which becomes more altered as fatigue symptoms worsen, the researchers believe that the symptom of fatigue can be ameliorated. The choice of the beta frequency band was based on its involvement in motor processing and its potential relevance to the experience of fatigue. Additionally, the researchers considered that training in the alpha and/or beta bands might yield better results compared to slow delta or high gamma bands, as the latter can be affected by eye artifacts, which are common during visual tasks involved in EEG-Neurofeedback training (Buyukturkoglu et al., 2017).

With that, the studies in this section call attention to the variety of methods and techniques used for analyzing EEG signals collected at a resting state. Methods and techniques, such as IMF selection methods, recurrence quantification analysis (RQA), coherence analysis, mutual information, and mutual information, were all used in their respective experiments to extract nonlinear features and evaluate the functional connectivity in MS patients. These studies show how these measures can be used for detecting changes caused by MS in EEG signals and recognizing signs of cognitive dysfunction, neurological impairment, and information processing deficits accompanied by MS. The variety in the findings highlights the importance of identifying the appropriate analysis to understand the EEG activity in subjects with MS.

#### Task-based experiments

Raeisi et al. (2020) used an extension of bivariate empirical mode decomposition (BEMD) to analyze a task-based experiment (three visual stimuli were used to distinguish MS from healthy groups in the study). Each possible pair of EEG recordings was pre-processed to extract five pairs of IMFs by the bivariate empirical mode decomposition (BEMD) method. Phase synchrony between each pair of IMFs was calculated utilizing the mean phase coherence (MFC) method and Hilbert transform. To classify healthy and MS subjects, ReliefF and KNN classification methods were applied. Based on the results, the accuracy, sensitivity, and specificity of the red-green task were 93.09, 91.07, and 95.24%, respectively, while those of the black-white task were 90.44, 88.39, and 92.62%, and those of the blue-yellow task were 87.44, 87.05, and 87.86%. The experimental results indicated that the method could be generalized to automate MS diagnosis systems (Raeisi et al., 2020).

Karacan et al. (2022) focused on classifying the environment according to EEG signals. A cognitive task was performed on a computer by healthy volunteers and the MS patient group, and then the same task was performed in a virtual reality environment. After extracting chaotic entropies and fractal dimensions, the accuracies of different classification methods were compared. A KNN classifier performed best for volunteers with MS with an accuracy of 95.45%. Additionally, after inducing photic stimulation in different EEG subbands [delta (1–4 Hz), theta (4–8 Hz), alpha (8–13 Hz), and beta (13–30 Hz)] and feature extraction, the proposed ensemble subspace KNN classifier algorithm performed the automatic classification of MS with an accuracy of 80%, a sensitivity of 72.7%, a specificity of 88.9%, and a positive predictive value of 88.9%. The authors proposed using photonic stimulation EEGs for the pre-diagnosis of MS (Karacan et al., 2022).

The nonlinear model proposed by Torabi et al. (2017) classified healthy individuals and individuals with MS into two groups. The authors used the nonlinear features of EEG signals to distinguish healthy volunteers and MS patients performing a color-and-luminance-changing task and a direction-changing task. The Katz fractal dimension was the most “informative nonlinear feature” when combined with an SVM. Their model achieved high classification performances of 93.08 and 79.79% for the direction-based and the color-luminance-based tasks, respectively (Torabi et al., 2017).

Porcaro et al. (2019) sought an alternative treatment method to address fatigue brought upon by the side effects MS patients experience with pharmaceutical treatments. They carried out a five-day transcranial direct current stimulation (tDCS) focusing on the somatosensory representation of the body (S1), known as FaReMuS treatment. Utilizing fractal dimension and mutual information, 48% of the variance of fatigue was explained, paving the way for personalized neuromodulation techniques that can be used to address fatigue in patients with MS. The authors observed that the left side of the whole-body somatosensory area (S1) showed a significant change after neuromodulation. The nonlinear analyses helped demonstrate that left S1 was impaired in MS patients prior to treatment, and the difference between S1 in MS patients vs. healthy patients disappeared after treatment, which is a significant step toward identifying an improved treatment (Porcaro et al., 2019).

Tomasevic et al. (2013) The findings of this study, in terms of coherence analysis, indicate that indices related to movement execution are significantly associated with fatigue rather than morpho-structural measures related to the primary sensorimotor network. Specifically, fatigued patients exhibited cortico-muscular coupling at faster frequencies and corrected the pressure exerted during handgrip at higher frequencies. The disruption of primary somatosensory network patterning in MS indicates that intra-cortical synchronization phenomena affecting cortico-muscular coupling also play a significant role in motor control. The study suggests that the quality of communication between the cortex and muscles is impaired in fatigue, while the spectral features of the motor cortex and muscular oscillatory activities remain unaltered (Tomasevic et al., 2013).

The studies in this section underscore the diverse array of methods and techniques used for analyzing EEG signals collected while participants completed a task. The studies provide valuable insight into the growing body of knowledge regarding using EEG in the diagnosis, treatment, and understanding of MS. EEG-based techniques provide essential tools for academics, physicians, and patients in the pursuit of better management and care for people with MS by using modern signal processing, nonlinear analysis, and creative intervention strategies.

### Limitations of the studies in this review

The selected studies are subject to several limitations. The sample sizes of some were relatively small (Ahmadi and Pechenizkiy, 2016; Carrubba et al., 2019; Karaca et al., 2021). Furthermore, several studies did not have a diverse participant group (e.g., all females). The results may differ if males were included in the study (Carrubba et al., 2010). Another limitation that we identified in the selected studies was the lack of physical exertion in task-based experiments. Examining chaotic data while a patient with MS performs an exerting physical task may provide additional unique results that may help further understand the complexity of brainwave activity in patients with MS. However, it is crucial to acknowledge the potential challenges associated with inducing artifacts during physical task experiments and the need for meticulous methodological considerations (Jiang et al., 2019). To address this, future studies incorporating physically exerting tasks should apply appropriate pre-processing techniques to minimize artifacts, such as independent component analysis (ICA)-based algorithms, regression, adaptive filter, common component analysis (CCA), or combinations of other methods tailored to the specific artifacts induced by the physical tasks (Jiang et al., 2019). These methods have shown promise in dealing with various artifacts encountered in EEG recordings, including muscle artifacts. Additionally, prior to the physical task, participants should be adequately prepared and familiarized with the experimental setup through clear instructions and practice sessions, reducing potential sources of artifacts related to task execution. Therefore, the findings of future studies could be strengthened by including larger numbers of participants from different groups and by them performing various physical tasks.

## Conclusion

The aim of this review was to provide an extensive overview of existing knowledge on the use of nonlinear dynamics in analyzing the EEG results of MS patients. In the selected studies, chaos in brainwave activity as measured with an EEG was explored in both the resting-state and during task-based experiments. In the task-based experiments, participants were asked to perform attention, vision, and cognition tasks to determine how such activities affect people with MS vs. people without. In the resting-state experiments, participants were at rest with no stimuli present. The authors of the studies were able to examine the similarities and differences between both sets of participants with several different nonlinear analyses. Wavelets, EMD, entropy, and RQA were some of the most commonly used nonlinear methods used to make sense of the nonlinear data acquired. Thus, there are numerous ways in which chaos theory techniques have assisted EEG researchers in understanding, diagnosing, and treating MS, and these are summarized below.

A decrease in mutual information between brain areas provided researchers with an indicator of MS patients’ processing deficits, and HVG captured key features of systems via the synchronization matrix of the brain network. Both analyses provided results that helped researchers better understand MS and inform further (Ahmadi and Pechenizkiy, 2016). There are several promising methods for diagnosing MS, with RQA and machine learning algorithms being notable (Carrubba et al., 2010, 2019). For example, the quantifier value %R (percent recurrence) is increased in MS patients compared with healthy patients, indicating its potential as a possible indicator of MS (Carrubba et al., 2012). Additionally, the model proposed by Mohseni and Moghaddasi (2022) utilizing nonlinear feature extraction and classification demonstrated promising results for diagnosing MS (Mohseni and Moghaddasi, 2022). Machine learning algorithms can enable the search and analysis of large datasets about potential biomarkers to help find clinically useful courses of treatment (Hossain et al., 2022). Furthermore, to avoid the negative side effects associated with pharmaceutical treatments, Porcaro et al. (2019) utilized fractal dimension and mutual information to better understand fatigue with the hope of developing improved treatments (Porcaro et al., 2019).

Multiple sclerosis (MS) studies can be clinically translated into improved diagnostic tools, advanced treatments, assistive devices, etc. However, chaotic analysis is not just applicable to MS; it can detect seizures in epileptic patients, diagnose Alzheimer’s disease, and provide beneficial information for other neurological disorders, all of which demonstrate the potential for nonlinear analysis of chaos to be highly effective in the medical profession (Jacob and Gopakumar, 2018).

## Data availability statement

The original contributions presented in this study are included in the article/Supplementary material, further inquiries can be directed to the corresponding author.

## Author contributions

CH and SK conducted the literature search, directed manuscript preparation, revisions, and editing. SH provided critical feedback and helped improve the overall organization of information. WK conceived the study conception, evaluated the screening, methodological quality of the literature search and supervised the final intellectual content. All authors contributed to the article and approved the submitted version.
