# Supplementary material for: Examining electroencephalogram signatures of people with multiple sclerosis using a nonlinear dynamics approach: a systematic review and bibliographic analysis
Source: Front Comput Neurosci. 2023 Jun 29;17:1207067. doi: 10.3389/fncom.2023.1207067 (PMC10344458; doi:10.3389/fncom.2023.1207067)
Supplement: Supplementary file 1 [file Table_1.pdf]

Supplementary Material

Examining Electroencephalogram Signatures of People with Multiple Sclerosis using a Nonlinear Dynamics Approach: A Systematic Review and Bibliographic Analysis

Christopher Ivan Hernandez[1*†], Shaida Kargarnovin[1†], Sara Hejazi[1], and Waldemar Karwowski[1]

[1]Computational Neuroergonomics Laboratory, Department of Industrial Engineering and Management Systems, University of Central Florida, Orlando, FL 32816, USA

***Correspondence:**
Christopher Ivan Hernandez
c.hernandez@knights.ucf.edu

†These authors contributed equally to this work and share first authorship

1 Supplementary Tables

Supplementary Table 1. MS studies using nonlinear dynamical methods

Study	Category	Nonlinear Analysis	Participants	EEG Channels	Experiment	Summary of Findings
(Ahmadi and Pechenizkiy)	Task-Based	Horizontal Visibility Graph	Total: 2<br>• 1 MS subject<br>-Male<br>-51 years<br>• 1 healthy subject	16 channels	Participants selected images (out of six) based on the application scenario.	The HVG method performed well compared to other VG-based models and traditional synchronization measures. HVG demonstrated encouraging results for analyzing chaotic data and neural systems with weak coupling.

(Torabi, Daliri and Sabzposhan, 2017)	Task-Based	Lyapunov Exponents, Approximate & Sample Entropy, Hurst Exponent, Fractal Dimension, & L-Z Complexity	Total: 12<br>• 5 MS subjects<br>• 7 healthy subjects<br>• 4 males and 8 females<br>• Age: 25-45 years	14 channels	Two experiments (attention task): color and luminance (feature) changing task and direction (position) changing task	There is a correlation between the front lobe function and MS disease since the front lobe plays a large role in concentration and decision-making. Also, beta waves are connected to concentration - they were the most affected by MS.
(Carrubba et al., 2012)	Resting-State	Recurrence Quantification Analysis	Total: 20<br>• 10 MS subjects<br>-All females<br>-Age: 18-52<br>• 10 healthy subjects<br>-All females<br>-Age: 25-53 years	6 channels	Subjects sat in a dark room in a comfortable chair with their eyes closed while data was collected	There was an expectation for there to be an increase in percent recurrence (%R) and percent determinism (%D). The authors examined an increase in %R in MS patients and no difference in %D in MS patients compared to healthy subjects, suggesting that %R is an indicator of nonlinearity. %R and %D are possible baselines to diagnose MS because they reveal evoked potentials.
(Mohseni and Moghaddasi, 2022)	Resting-State	Sample Entropy, Higuchi Fractal Dimension, & Katz Fractal Dimension	Total: 40<br>• 19 MS subjects<br>-14 females and 5 males<br>• 21 healthy subjects	5 channels	The EEG signal recordings were obtained under different protocols at a resting state with eyes closed.	Using wavelet analysis techniques, they divided all five sub-bands of EEG signals, performed signal windowing, and extracted efficient features from the data. Additionally, the signal's alpha, beta, and gamma bands are important, and the accuracy, sensitivity, and specificity levels are

			-9 females and 12 males<br><br>• Avg. age: 28.4 ± 5.2			higher than 98.5%. Compared to similar MS diagnostic methods, the proposed method achieved significantly higher diagnostic accuracy.
(Kotan et al.)	Resting-State	Empirical Mode Decomposition (EMD), Hurst exponent, & Higuchi Fractal Dimension	Total: 100<br><br>• 50 MS subjects<br><br>• 50 healthy subjects	21 channels	Not mentioned	This study compares three methods for selecting IMF for EEG signals; power-based, correlation-based, and power-spectral density-based combined with Hurst exponent and Higuchi fractal dimensions. Then, three classifiers were used, K-nearest neighbors, multilayer perceptron neural networks, and random forest. It was concluded that IMF selection methods have a variable effect on accuracy based on classifier selection.
(Karaca, Akşahin and Öcal, 2021)	Task-Based	Wavelet Transform	Total: 20<br><br>• 9 MS subjects<br><br>-All males<br><br>• 11 healthy subjects<br><br>-All males<br><br>• Age: 20 - 50	19 channels	EEG was recorded in a dark room under intermittent photic stimulation at 5, 10, 15, 20, and 25 Hz. Stimulation lasted 20 seconds.	In order to evaluate whether EEG is affected by photic stimulation in MS and healthy controls, EEG signals were recorded at 5 Hz, 10 Hz, 15 Hz, 20 Hz, and 25 Hz from MS and healthy controls. The proposed ensemble subspace k-NN classifier algorithm had an accuracy of 80 %, a sensitivity of 72.7 %, a specificity of 88.9 %, and a positive predictive value of 88.9 %. In conclusion, a pre-

						diagnosis of MS can be made using photonic stimulation electroencephalogram signals.
(Karacan et al.)	Task-Based	Wavelet Transform	Total: 39<br>• 11 MS subjects<br>• 28 healthy subjects	32 channels	Subjects performed a cognitive task measuring mathematical and working ability in a computer environment and with an Oculus Quest 2 virtual reality headset.	A cognitive task was performed by healthy volunteers and the MS patient group on a computer, and then the same task was performed in a virtual reality environment. After extracting chaotic entropies and fractal dimensions, the accuracy of the different classification methods was compared. A KNN classifier performed best for volunteers with MS with an accuracy of 95.45%.
(Carrubba, Frilot and Marino, 2019)	Task-Based	Recurrence Quantification Analysis (RQA)	Total: 20<br>• 10 MS subjects<br>-All females<br>-Age: 18-52 years<br>• 10 healthy subjects<br>-All females<br>-Age: 24-53 years	6 channels	Subjects sat in a darkened room with their eyes closed on a comfortable chair for 10 minutes.	The optimal embedding dimension and time delay of 5 points maximize RQA's ability to detect deterministic activity in EEG time series. In the MS patients, the RQA quantifier values were significantly higher than those in the healthy controls, indicating the disease is associated with detectable changes in the EEG.

(Raeisi et al., 2020)	Resting-State	Bivariate Empirical Mode Decompositions (BEMD) & Phase Coherence	Total: 31<br>• 16 MS subjects<br>-10 females and 6 males<br>• 15 healthy subjects	32 channels	A total of three types of visual stimuli were used in the experiment, including red-green, blue-yellow, and black-white.	Three visual stimuli were used to distinguish MS from healthy groups in this study. Based on these results, the accuracy, sensitivity, and specificity of the red-green task were 93.09%, 91.07%, and 95.24%, respectively, while those of the black-white task were 90.44%, 88.39%, and 92.62%, and those of the blue-yellow task were 87.44%, 87.05%, and 87.86%.
(Lenne et al., 2013)	Task-Based	Coherence Function & Mutual Information	Total: 51<br>• 31 MS subjects<br>-18 females and 13 males<br>-Avg. age: 39.5 ± 9.5<br>• 20 healthy subjects<br>-14 females and 6 males<br>-Avg. age: 40.5± 11.5	17 channels	Twenty minutes of EEG recordings were performed in eyes closed and resting conditions. Two one-hour sessions spaced by no more than one month were conducted.	A significant decrease in mutual information in a network of brain areas was observed as the main outcome of cortical communication impairment in MS. The findings suggest that averaged inter-hemispheric mutual information acquired in resting state conditions is a potential indicator for the neurological dysfunction observed in patients with MS.
(Carrubba et al., 2010)	Resting-State	Recurrence Quantification Analysis & Nonlinear Mathematical Algorithm	Total: 31<br>• 11 MS subjects<br>-All females<br>-Age: 18-52 years	6 channels	The participants were exposed (eyes closed) in an isolation chamber to reduce the effect of	The authors tested whether cognitive processing would change in MS when they elicited a subliminal stimulus to patients with MS and patients without. Onset response occurred in 27% of patients with MS and 85% in subjects without

			• 20 healthy subjects (two gender- and aged-matched groups)<br><br>-Group 1: All females<br><br>-Group 1 Age: 19 – 52 years<br><br>-Group 2: All females<br><br>-Group 2 Age: 24 – 53 years		random ambient stimuli.	MS. The method outlined in the study enabled an evaluation of the degree of synchronization between brain networks, which is exactly the high-level brain function they believed to be affected.
(Porcaro et al., 2019)	Task-Based	Fractal Dimension and Mutual Information	Total: 30<br><br>• 18 MS subjects<br><br>-4 males and 14 females<br><br>• 12 healthy subjects<br><br>-3 males and 9 females)	64 channels	Subjects executed a handgrip on an air-bulb that was giving resistance with the left and right hands separately.	Using EEG, the authors were able to see that the left side of whole-body somatosensory area (S1) had a significant change after neuromodulation. Fractal dimension is a good tool for investigating brain network functionality. Fractal dimension demonstrated that left S1 was impaired in MS patients prior to treatment, and the difference between S1 in MS patients vs. healthy patients disappeared after treatment.

(Tramonti et al., 2018)	Resting-State	Weighted Symbolic Mutual Information (wSMI)	Total: 16<br>• 16 MS subjects<br>-6 males and 10 females<br>-Age: 27-67 years	64 channels	Participants volunteered in a task-oriented circuit training (TOCT) for gait rehabilitation, and their motor performance was assessed and analyzed. Resting-state EEG was calculated before and after the gait rehabilitation	EEG-based-connectivity measures were effective in tracking functional recovery after gait rehabilitation (TOCT). The nonlinear analysis, weighted symbolic mutual information (wSMI), was used to evaluate non-random joint fluctuations between two EEG signals, and it demonstrated strong results for understanding behavioral changes after gait rehabilitation. The nonlinear analysis showed that phase synchronization is correlated with functional recovery.
(Leocani et al., 2000)	Resting-State	Coherence Analysis	Total: 50<br>• 28 MS subjects<br>-11 females and 17 males<br>-Age: 20 – 63 years<br>• 22 healthy subjects<br>-Similar sex and age distribution to MS subjects	19 EEG Channels	EEG was recorded at rest for 10 minutes with subjects' eyes closed.	A large reduction in coherence was seen in EEG activity between the anteroposterior and interhemispheric areas in cognitively impaired MS patients when compared to healthy subjects and cognitively normal MS patients. A reduction in coherence means fewer functional connections. The authors concluded that cognitive impairment in multiple sclerosis is contingent on the corticocortical connection connected to the demyelination and/or the axonal loss that lies beneath the cortex in the white matter.

(Jouzizadeh et al., 2021)	Resting-State	Coherence Analysis	Total: 68<br>• 34 MS patients<br>-18 females and 16 males<br>-Avg. age 27.32 ± 4.37 years<br>• 34 healthy patients<br>-18 females and 16 males<br>-Avg. age 25.12 ± 7.51 years	19 EEG Channels	5 min of EEG data were collected during a resting-state eyes open condition.	The authors concluded that the resting-state functional connectivity of MS patients compared to normal participants was different. It was also demonstrated that EEG has a potential for producing similar results to fMRI, and betweenness centrality and small-world propensity are effective indicators in differentiating an individual with MS from an individual without MS.
(Tomasevic et al., 2013)	Task-Based	Coherence Analysis	Total: 20<br>• All MS patients	23 EEG Channels	Data were collected during a weak handgrip task.	The findings of this study, in terms of coherence analysis, indicate that indices related to movement execution are significantly associated with fatigue rather than morpho-structural measures related to the primary sensorimotor network. Specifically, fatigued patients exhibited cortico-muscular coupling at faster frequencies and corrected the pressure exerted during handgrip at higher frequencies. The disruption of primary somatosensory network patterning in MS indicates that intra-cortical synchronization phenomena affecting

						cortico-muscular coupling also play a significant role in motor control. The study suggests that the quality of communication between the cortex and muscles is impaired in fatigue, while the spectral features of the motor cortex and muscular oscillatory activities remain unaltered.
(Buyukturkoglu et al., 2017)	Resting-State	Coherence Analysis	Total: 29<br><br>18 relapsing-remitting (RR) MS patients<br><br>-12 females<br><br>-Age range: 24–47 years<br><br>11 healthy people<br><br>-9 females<br><br>-Age range: 28–49 years	19 EEG Channels	The participants were seated in a relaxed position on chairs facing a screen that displayed a fixation point. Resting-state data was collected from each participant for a minimum of 4 minutes while their eyes remained open.	They discovered that a potential EEG-Neurofeedback system for MS fatigue should train patients to voluntarily decrease coherence in the beta frequency band between homologous temporoparietal cortices. By targeting and modulating this specific brain organization feature, which becomes more altered as fatigue symptoms worsen, the researchers believe that the symptom of fatigue can be ameliorated. The choice of the beta frequency band was based on its involvement in motor processing and its potential relevance to the experience of fatigue. Additionally, the researchers considered that training in the alpha and/or beta bands might yield better results compared to slow delta or high gamma bands, as the latter can be affected by eye artifacts, which are

						common during visual tasks involved in EEG-Neurofeedback training.
